# Long Read Alignment with Parallel MapReduce Cloud Platform

**DOI:** 10.1155/2015/807407

**Published:** 2015-12-29

**Authors:** Ahmed Abdulhakim Al-Absi, Dae-Ki Kang

**Affiliations:** ^1^Department of Ubiquitous IT, Graduate School, Dongseo University, 47 Jurye-ro, Sasang-gu, Busan 47011, Republic of Korea; ^2^Department of Computer & Information Engineering, Dongseo University, 47 Jurye-ro, Sasang-gu, Busan 47011, Republic of Korea

## Abstract

Genomic sequence alignment is an important technique to decode genome sequences in bioinformatics. Next-Generation Sequencing technologies produce genomic data of longer reads. Cloud platforms are adopted to address the problems arising from storage and analysis of large genomic data. Existing genes sequencing tools for cloud platforms predominantly consider short read gene sequences and adopt the Hadoop MapReduce framework for computation. However, serial execution of map and reduce phases is a problem in such systems. Therefore, in this paper, we introduce Burrows-Wheeler Aligner's Smith-Waterman Alignment on Parallel MapReduce (BWASW-PMR) cloud platform for long sequence alignment. The proposed cloud platform adopts a widely accepted and accurate BWA-SW algorithm for long sequence alignment. A custom MapReduce platform is developed to overcome the drawbacks of the Hadoop framework. A parallel execution strategy of the MapReduce phases and optimization of Smith-Waterman algorithm are considered. Performance evaluation results exhibit an average speed-up of 6.7 considering BWASW-PMR compared with the state-of-the-art Bwasw-Cloud. An average reduction of 30% in the map phase makespan is reported across all experiments comparing BWASW-PMR with Bwasw-Cloud. Optimization of Smith-Waterman results in reducing the execution time by 91.8%. The experimental study proves the efficiency of BWASW-PMR for aligning long genomic sequences on cloud platforms.

## 1. Introduction

Bioinformatics involves the biological, genomic, statistics, mathematics, and computer science disciplines of study. Analysis of genomic and biological sequence data is one of the important parts of bioinformatics [1]. The analysis of genomic sequences enables us to understand the genetic structure, and its bases of drug response and disease. Numerous researchers who are working in this interdisciplinary field perform gene structure and functionality analysis in order to discover new gene sequences and understand gene origin. For the analysis purpose, existing genomic databases such as Google Genomics [2] and NCBI [3] (generally in 100's of GB in size) are used to identify the similarities. Identification of similarities/dissimilarities is achieved by sequence comparison algorithms. Comparisons of biological sequences produce matching alignments and similarity scores. These similarity scores represent the similarities/dissimilarities between the considered biological sequences. The matching alignments and similarity scores are used for secondary structure predictions and multiple sequence alignments which are highly complex operations that rely on the accuracy of the comparison algorithm used. Applications related to cancer research, forensics, agrigenomics, genetic disease identification, microbial research, reproductive health, human whole-genome sequencing, and many more rely on sequence alignment algorithms for analysis.

Genomic sequencing data is obtained from the developed Next-Generation Sequencing (NGS) technologies (e.g., from Illumina, Solexa, and Pacific Bioscience). Most of the genomic data available consists of millions of genomic sequences of short reads [4]. With the advances in sequencing technologies, millions of sequences with greater read lengths >1000 bp are now being generated. Based on genomic data, the sequencing tools can be basically classified into two categories:

(1) Short read aligners: used to align genomic sequences whose read length is between 32 bp and 200 bp, that is, BWA [4] and Bowtie2 [5].
(2) Long read aligners: used to align genomic sequences whose length is greater than 1000 bp, that is, Illumina.

Examples of short read aligners include FASTA [6], BLAST [7], BLAT [8], SOAP [9], and Burrows-Wheeler Aligner (BWA) [4]. For long read alignment, researchers have proposed Burrows-Wheeler Aligner's Smith-Waterman (BWA-SW) Alignment [10], Bowtie2 [5], Cushaw2 [11], and BWA-MEM [12].

Existing bioinformatics applications require both management of huge amounts of data and heavy computation for analysis. Nearly 3 billion US dollars and 10 years were required to produce the initial human reference genome containing about 3.5 billion base pairs. The latest developments in sequencing technologies available produce enormous amount of data in terms of gigabytes per run [13]. In NGS large sample size applications, users are required to wait for sufficient resources to become available in which the time needed to complete processing becomes unpredictable. Analyzing such enormous amount of data and its storage are problems that exist. To address these high computation needs, grid or cluster computing platforms have been provided to researchers [14]. The provided grid or cluster computation platforms are constrained by hardware capacity and concurrent access capacity to support multiple users. The ever growing gap between computing capabilities and sequencing throughput is presented in [15].

Using cloud platforms, one can solve the storage and computing problems that exist in gene sequencing. Cloud computing platforms provide flexibility, scalability, and on-demand access to resources. Moreover, adoption of cloud computing technologies eliminates the costs incurred in establishing, maintaining large physical storage systems, and computing clusters. Cloud users pay for the utilized storage and computing resources without worrying about maintenance, availability, and reliability related issues. Although cloud computing provides scalable and flexible infrastructure, parallel computing models on cloud platforms/infrastructure are required to achieve the desired goal of analyzing gene sequences. One such framework for the cloud, called MapReduce model [16], was introduced by Google. Another model developed by University of California at Berkeley proposed the Spark platform [17] for cloud computing. The Pregel framework for cloud environments is presented in [18]. A comprehensive survey of most recent research and development in cloud-based service computing solutions in research of genome informatics is available in [19, 20]. In research of genomic analysis, there are a number of cloud computing providers that offer cloud-based services solutions such as Google Cloud Genomics [3], Amazon [21], and Microsoft Azure [22]. The necessity for cloud computing for genomic analysis has been well discussed by leaders in bioinformatics and computational biology [23]. Galaxy [24] is a powerful web-based system for performing genome analysis tasks and one of many models that manage bioinformatics databases. Galaxy built on Amazon's Elastic Compute Cloud (EC2) with CloudMan framework to assist researchers executes their own Galaxy server on cloud infrastructure. However, Galaxy has data storage and data manipulation bottlenecks for large datasets with capability of analyzing one sample at a time and does not utilize the elastic cloud compute capabilities completely. This drawback is caused by its dependence on a single shared file system. A significant bottleneck has been reported in [25] when processing large datasets across distributed compute resources. Among all the cloud computing platforms available, Hadoop MapReduce is by far the most popular choice due to its ease to tune, open source nature, and acceptability by industry and academic organizations. In order to utilize the full potential of cloud infrastructure, public cloud service providers offer virtualized resources in terms of both hardware and software [26]. The virtualization enables user specific customization, flexibility, application execution environments, and cost efficiency and minimizes power consumption [27, 28].

**Figure 1:**
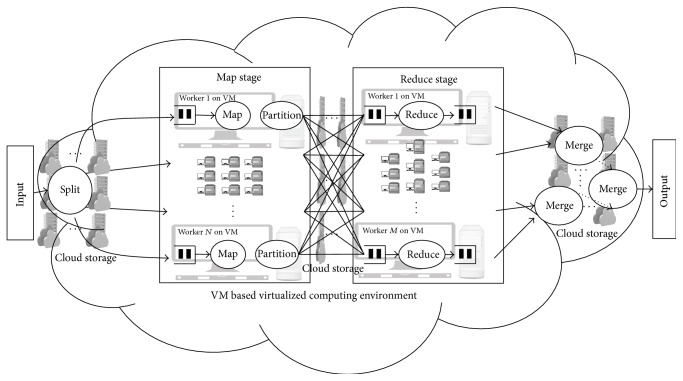
MapReduce model deployed using VM based computing environment.

The Hadoop framework [29] adopts a MapReduce model for computing a user specific application on cloud platforms. In the MapReduce model, a dual phase execution approach is adopted. In the initial phase, input data to be processed is split into chunks. Each chunk is associated with a mapper or a map worker that provides 〈Key∣Value〉 pairs as outputs. The outputs values are sorted on the basis of the Key values associated. The sorted values are provided to reduce workers, that is, 〈Key∣SortedList(Value)〉. Reduce workers store the results in Hadoop distributed file system. The map and reduce workers are generally virtual machines (VMs) in public cloud environments. A simple MapReduce model deployed on the VM based computing environment is shown in Figure 1.

Next-Generation Sequencing (NGS) tools like CloudAligner [13], Cloud Burst [30], SeqMapReduce [31], and Crossbow [32] adopt the Hadoop framework. The major drawback of these alignment tools is that they are short read aligners. The short read aligners prove to be efficient when single-gap or ungapped alignment is to be computed. Considering long reads, these alignment algorithms exhibit performance degradation and affect accuracy proved by the results presented in [10]. For alignment considering long sequence reads, optimization of the BLAST algorithm and its deployment on Hadoop platform is presented in [33]. For long read aligners, the BWA-SW algorithm in [10] has been found to be efficient and suitable. In [34], the Bwasw-Cloud algorithm considering Hadoop platform is presented. The Bwasw-Cloud adopts BWA-SW algorithm for alignment. Based on the literature reviewed, it is evident that limited work has been carried out considering long read aligners required to support analysis of genomic data generated from current and future sequencing technologies. There is an increasingly urgent need to provide a reliable and scalable support for the ever-increasing convergence of NGS data. However, there is clearly a lack of standardized and affordable NGS management solutions on the cloud to support the growing needs of translational genomics research [20]. This is a major motivating factor for the authors of this paper.

All the existing long read aligners for cloud environments consider the Hadoop framework. Genome alignment is an iterative process and Hadoop incurs considerable performance overheads when iterative applications are hosted on the framework [18, 35]. The aligners presented in [33, 34] need to rely on multiway joins as genomic sequences are split and needed to be merged prior to the reduce phase. The performance of Hadoop suffers when multiway joins are considered [36]. The Hadoop framework considers sequential processing of the map followed by reduce stage that affects performance [37].

Therefore, in this paper, we present Burrows-Wheeler Aligner's Smith-Waterman Alignment on Parallel MapReduce (BWASW-PMR) to perform long read alignments on a cloud platform. BWASW-PMR adopts the BWA-SW presented in [10] to perform long read alignments of genomic data. MapReduce model is considered for the execution on the public cloud environment. The Smith-Waterman (SW) algorithm [38, 39] in BWA-SW is optimized using parallel computation technique. To overcome the drawbacks of Hadoop MapReduce, a parallel execution strategy of the map and reduce workers is considered in BWASW-PMR. The map and reduce functions executions on the VM based worker nodes are parallelized to reduce execution time. The aligner presented in [34] bears the closest similarity to the BWASW-PMR proposed and is considered for comparison. The major contribution of our work can be summarized here:

- Optimization of Smith-Waterman in the BWA-SW long read sequence alignment.
- A custom MapReduce framework to support the required computations for long sequence alignment.
- Parallel map and reduce workers execution strategy.
- Parallel execution of the map and reduce functions at worker nodes.

This paper is organized as follows.  Section 2 presents related work background.  Section 3 discusses the proposed Burrows-Wheeler Aligner's Smith-Waterman Alignment on the Parallel MapReduce, BWASW-PMR. The SW algorithm and its considered optimization are also presented in Section 3. The results and the experimental study are shown in Section 4. Comparison notes with related work counterparts are discussed in the penultimate section. The concluding remarks and future work are discussed in the last section.

## 2. Background

### 2.1. Smith-Waterman (SW) Algorithm

Burrows-Wheeler Aligner's Smith-Waterman (BWA-SW) Alignment relies on SW algorithm to align the seed matches of sequences. Let *q*_0_ and *q*_1_ represent two genomic sequences obtained from the seed matches. The Smith-Waterman algorithm computes the similarity matrix score initially. Using the similarity matrix and backtracking technique, optimal alignment is obtained. Let *𝒳* represent the size of *q*_0_ and *𝒴* represents the size of *q*_1_. For sequence *q*_0_, there are *𝒳* + 1 possible prefixes and, for *q*_1_, there are *𝒴* + 1 possible prefixes including the empty sequence. Let *q*_seq_[1,…, *𝒴*] denote a prefix of *𝒴* characters and *q*_seq_[*𝒳*] represents the *𝒳*th character of a sequence *q*_seq_. The similarity score between prefixes *q*_0_[1,…, *a*] and *q*_1_[1,…, *b*] is represented as *q*_*a*,*b*_. The similarity matrix is denoted by *Z* and its size is (*𝒳* + 1, *𝒴* + 1). The first row and column of *Z* are initialized to 0. The remaining elements of *Z* (indexed by (*a*, *b*)) are computed using

(1) $$\begin{array}{c} Z_{a,b}=\max\left\{\begin{array}{cc} 0 & \\ I_{a,b} & \\ J_{a,b} & \\ Z_{a-1,b-1}+R\left(a,b\right), & \end{array}\right. \end{array}$$

where *R*(*a*, *b*) is a function providing values of exact match or mismatch; that is, if *q*_0_[*a*] = *q*_1_[*b*] then *R*(*a*, *b*) = *X*_*i*_ (identical characters among sequences *q*_0_ and *q*_1_); otherwise, *R*(*a*, *b*) = *M*_*i*_  (if characters among sequences *q*_0_ and *q*_1_ are unique). *I* and *J* represent the matrices in accordance with the affine gap model. The matrices *I* and  *J* are used to determine the gaps and are defined as

(2) Ia,b=max⁡Ia,b−1−GEZa,b−1−GF,Ja,b=max⁡Ja−1,b−GEZa−1,b−GF,

where *G*_*E*_ and *G*_*F*_ represent the first and successive gap penalty.

Backtracking algorithm is used to find the optimal alignment between *q*_0_ and *q*_1_. The backtracking begins with a cell in *Z* that holds the highest score and proceeds till a zero valued cell is reached. Smith-Waterman algorithm provides optimal alignments and similarity scores. It must be noted that computation of matrix *Z* is bounded by the running time of the slowest *Z* task due to the dependencies it exhibits.

### 2.2. Burrows-Wheeler Aligner's Smith-Waterman (BWA-SW) Alignment Algorithm

**Figure 2:**
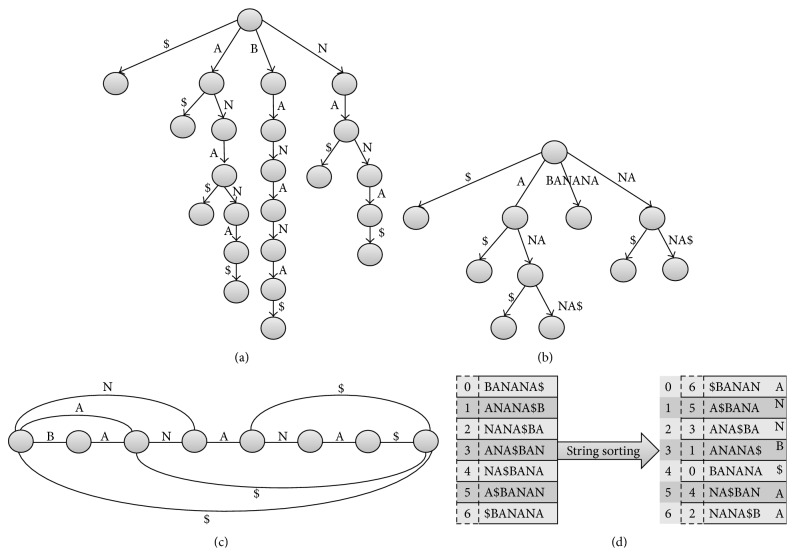
Example of reference in a prefix trie, query in prefix DAWG, and the suffix array of the input string “BANANA$”. (a) represents reference prefix trie searching for any substring of the string by staring at the root and following matches down the tree until being exhausted. (b) represents prefix DAWG constructed by collapsing nodes with the identical suffix array interval. DAWG transformed from the prefix trie of the query sequence. (c) represents a constructed string suffix automaton (DAWG). (d) represents the suffix array for string “BANANA$”. The dollar sign $ is a regular expression that denotes the end of a line in the reference sequence.

BWA-SW algorithm constructs a full-text index in minute space (FM-index) [40] of the query sequence *Q* and the reference sequence *R*. A prefix directed acyclic word graph (*Prefix* *DAWG*) is built using sequence *Q*. *prefix* *trie* is built using the sequence *R*. The prefix trie is a tree representation of sequence *R*. The tree is constructed by concatenating all edge symbols from any node in the graph as a route to the root node providing a unique string. The unique string obtained is a substring of the sequence *R*. Each node of the tree is represented using *suffix* *array* *interval*. Traversing from nodes generates strings that are lexicographically sorted. A node in *prefix* *trie* represents a string. The *suffix* *array* *interval*  of the node and the string it represents are considered to be equivalent to each other. From *prefix* *trie*, nodes exhibiting identical or similar *suffix* *array* *interval* are collapsed to form *Prefix* *DAWG*. Each node of *Prefix* *DAWG* established represents one or more substrings of *R*.  Figure 2 represents *prefix* *trie*, *Prefix* *DAWG*, and *suffix* *array* of the input string “BANANA$”.

Let PT(*X*) and DG(*X*) represent two functions that are used to form *prefix* *trie* and *Prefix* *DAWG* graph. In the BWA-SA algorithm, PT(*R*) and DG(*Q*) are initially computed. Let *a* be the root node of PT(*R*), and *b* represents the root node of DG(*Q*). The best score between the sequences *Q* and *R* is computed using a dynamic programming mechanism. Initialize *E*_*ab*_ = *F*_*ab*_ = *K*_*ab*_ = null considering the root nodes of PT(*R*) and DG(*Q*). For each of the parents, that is, *a*_*p*_, of the *a*th node in DG(*Q*), computation of *F*_*ab*∣*a*_*p*__, *K*_*ab*∣*a*_*p*__, and *E*_*ab*∣*a*_*p*__ is considered. The computation of *F*_*ab*∣*a*_*p*__ is

(3) $$\begin{array}{c} F_{ab\;|\;a_{p}}=\underset{}{\max}\left\{\;F_{a_{p}b},E_{a_{p}b}-g^{o}\;\right\}-g^{e}, \end{array}$$

where *g* ^*o*^ is the gap open penalty and *g* ^*e*^ is the gap extension penalty. *K*_*ab*∣*a*_*p*__ is computed using

(4) $$\begin{array}{c} K_{ab\;|\;a_{p}}=\underset{}{\max}\left\{\;K_{ab_{p}},E_{ab_{p}}-g^{o}\;\right\}-g^{e}, \end{array}$$

where *b*_*p*_ is the parent of the *b*th node in PT(*R*). The computation of *E*_*ab*∣*a*_*p*__ is achieved using

(5) $$\begin{array}{c} E_{ab\;|\;a_{p}}=\max\left\{\;E_{a_{p}b_{p}}+O\left(\;a_{p},a;b_{p},b\;\right),F_{a_{p}b_{p}},K_{a_{p}b_{p}},0\;\right\}, \end{array}$$

where *O*(*a*_*p*_, *a*; *b*_*p*_, *b*) represents the score between the symbol on edge (*a*_*p*_, *a*) and (*b*_*p*_, *b*). The computation of *E*_*ab*_, *F*_*ab*_, and *K*_*ab*_ is defined as

(6) Eab,Fab,Kab=Eab ∣ a′,Fab ∣ a′,Kab ∣ a′,When  Eab ∣ a′>0,−∞,−∞,−∞,all  other  cases,

where *a*′ = arg⁡max_*a*_*p*_∈*ar*(*a*)_⁡*E*_*ab*∣*a*_*p*__ and *ar*(*a*) is a set containing parent nodes of *a*. Variable *E*_*ab*_ represents the best matching score between the substrings, that is, substring *a* and substring *b*. Consider *E*_*ab*_ > 0 when the substrings *b* and *a* match. It is known that the Smith-Waterman algorithm provides accurate alignment results but requires large computation time. To optimize the computations, the reverse postorder traversal scheme on DG(*X*) and PT(*X*) is adopted. The dynamic programming mechanism in the BWA-SW algorithm enables identifying the seed matches of the genomic sequences. Based on the partial matches, the Smith-Waterman Alignment is considered to the extended matches. *seed* *interval* *pair*, that is, (*a*, *b*), is formed when the best score, that is, *E*_*ab*_, is high and *suffix* *array* *interval* size of *b* is within the threshold set. The seed matches are derived from *seed* *interval* *pairs* by analyzing the suffix array of sequences *Q* and *R*. Matching of the extended seed matches using Smith-Waterman algorithm is considered only when high matching scores are observed.

## 3. Proposed Burrows-Wheeler Aligner's Smith-Waterman Alignment on the Parallel MapReduce, BWASW-PMR

BWASW-PMR provides a cloud platform to perform long read alignments considering genomic data obtained from NGS techniques. The BWASW-PMR adopts the MapReduce computation model for cloud computation. To support scalability in BWASW-PMR, map and reduce worker nodes are deployed on a cloud cluster consisting of VMs. For long read alignments, the BWA-SW algorithm is adopted. The advantages of adopting the BWA-SW algorithm for long read alignments and its advantages over the existing aligners are found in [10]. The BWASW-PMR considers the genomic sequence alignment in dual phases, that is, map and reduce phase. Existing long read aligners for cloud platforms adopt the Hadoop framework. In the Hadoop framework based solutions, the map phase is executed and then the reduce phase is initiated. To overcome the drawbacks of Hadoop, a parallel execution strategy of the map and reduce phases is considered. Optimization of the Smith-Waterman algorithm is an additional feature considered in BWASW-PMR. Execution of the map and reduce functions is modelled to run in parallel utilizing all programming cores available in worker VMs.

### 3.1. Overview and Preliminaries

Let *R* represent a reference genomic sequence and *Q* the query sequence. BWASW-PMR is deployed on a cloud platform comprising a master node, map worker nodes, and reduce worker nodes. The master node of BWASW-PMR initializes *w* map and reduce worker nodes using virtual computing nodes. Each computing node or VM is assumed to have *p* CPU cores available for computation. Let *𝒯*_VM_Config_ represent the time taken to initialize the virtual computing platform. The sequence *R* is split into *R*′ chunks with overlapping sections (the overlapping sections are depicted in grey in Figure 2). The reference chunk and *Q* are sent to map worker nodes as input key, value pairs. The key value pairs considering the chunk *R*′ are represented as (*kr*, *vr*), where *kr* is a key and *vr* contains the overlapping offset data. The key value pair of *Q* is represented as (*kq*, *vq*), where *kq* is a key and *vq* is the query sequence. In each of the *w* map workers, sequence *Q* is further split into *Q*′ chunks and stored in the local memory available. Alignment is performed using the BWA-SW algorithm considering *R*′ and each *Q*′ in a parallel fashion utilizing the *p* cores. The Smith-Waterman algorithm in BWA-SW is optimized by a parallelization technique to reduce execution time. Let *𝒯*_MAP_ represent the average execution time of the *w* map worker nodes. The map workers after computation provide alignment locations between *Q* and chunk *R*′ along with the score. Multiple alignment locations and scores (i.e., *vm* along the chunk id of *R*′ and *km*) are stored in the temporary cloud memory as list(*km*, *vm*). The map function of BWASW-PMR can be defined as map((*kr*, *vr*), (*kq*, *vq*)) → list(*km*, *vm*). Reduce worker nodes *w* obtain intermediate data, that is, list(*km*, *vm*), to perform the shuffle and sort function. In the reduce phase, aggregation of all alignment locations, that is, list(*vd*), that are nonredundant and nonoverlapping is considered. The reduce operation can be defined as reduce(*km*, list(*vm*)) → list(*vd*). Let *𝒯*_REDUCE_ represent the average time required by *w* reduce worker nodes to perform the shuffle, sort, and reduce computation. The total makespan of the BWASW-PMR cloud platform to align the sequence *Q* against *R* is computed as

(7) $$\begin{array}{c} T=T_{VM\_Config}+T_{MAP}+T_{REDUCE}. \end{array}$$

**Figure 3:**
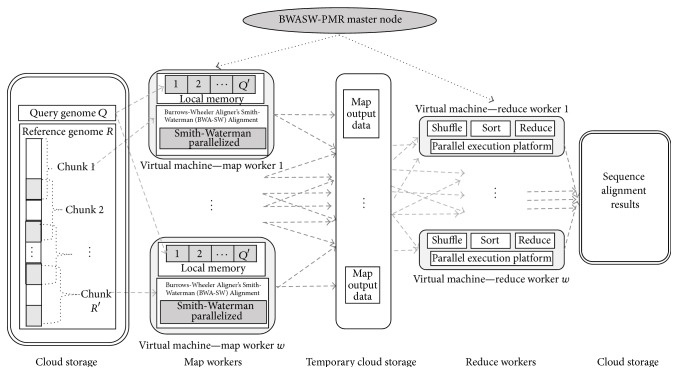
BWASW-PMR cloud model for long read sequence alignment.

The overview of the BWASW-PMR cloud platform is shown in Figure 3.

In the map phase of the BWASW-PMR cloud platform, alignment locations and corresponding scores between *Q* and chunk *R*′ are computed. The required data, that is, *Q* and chunk *R*′, is obtained from the cloud memory. Let *𝓉*_Get_Data_M_ represent the time taken to obtain the data. To reduce computation time using parallel computing techniques, the genomic sequence *Q* is split into *Q*′ chunks. Let *𝓉*_*QI*_ represent the time taken to split sequence *Q* into *Q*′ chunks. Sequence alignment considering one chunk of *Q*′ and *R*′ is performed using the BWA-SW algorithm. DG(*Q*′) and PT(*R*′) are initially constructed. The seed matches of sequences *Q*′ and *R*′ are computed using a dynamic programming mechanism. The seeds computed are extended to ensure rule alignment of the genomic sequences using the SW algorithm. To reduce computation time, parallelization of the SW algorithm is considered in BWASW-PMR. The parallelization technique to optimize computation is discussed in the latter subsection.

Let *𝓉*_BWASW_ represent the time taken to align *Q*′th chunk and *R*′ using the BWA-SW algorithm. The total time taken to align the total *Q*′ chunks is (*Q*′ × *𝓉*_BWASW_). As *p* computing cores are available with each worker node, the parallel computation of *p* number of chunks of *Q* is possible. The computation time considering all *Q*′ chunks and utilizing *p* cores is defined as ((*Q*′ × *𝓉*_BWASW_)/*p*). The alignment locations and scores obtained are stored in the cloud for reduce workers. The time taken to store this data per map worker is represented as *𝓉*_Store_Data_M_. The makespan of the *w*th map worker node can be defined as

(8) Tw_MAP=tGet_Data_M+tQI+Q′×tBWASWp+tStore_Data_M.

The average execution time of all the *w* map worker nodes is defined as

(9) $$\begin{array}{c} T_{MAP}=\frac{\sum_{i=1}^{w}T_{i\_MAP}}{w}. \end{array}$$

### 3.2. Reduce Phase

The master node in BWASW-PMR initializes *w* reduce worker nodes and *w* map worker nodes simultaneously. This parallel initialization mechanism enables reducing the total makespan *𝒯*. In the reduce phase, the shuffle function obtains the intermediate data (produced by the worker nodes) from the cloud storage. The alignment locations obtained from the intermediate data are sorted based on the offset data. The time taken by the *w*th reduce worker node to obtain the intermediate data and perform shuffle and sort operations is represented as *𝓉*_Get_Data_R_. The reduce function in BWASW-PMR is used to aggregate the alignment locations. The overlapping and redundant alignments are neglected. Parallelization of the reduce function is achieved by utilizing all *p* computing cores available with worker nodes. Let *𝓉*_Fn_R_/*p* represent the time taken to compute the reduce function utilizing the *p* computing cores. The reduce function provides the alignment results between sequences *R* and *Q* that are written to cloud storage for further analysis. Let *𝓉*_Store_Data_R_ represent the time taken by the *w*th reduce worker node to write alignment results into the cloud storage. The makespan of the *w*th reduce worker node can be defined as

(10) $$\begin{array}{c} T_{w\_REDUCE}=\left(\;t_{Get\_Data\_R}+\frac{t_{Fn\_R}}{p}+t_{Store\_Data\_R}\;\right). \end{array}$$

The average makespan of the *w* reduce worker nodes, that is, *𝒯*_REDUCE_, is defined as

(11) $$\begin{array}{c} T_{REDUCE}=\frac{\sum_{i=1}^{w}T_{i\_REDUCE}}{w}. \end{array}$$

Using (11) and (9), the total makespan of the BWASW-PMR can be defined as

(12) $$\begin{array}{c} T=T_{VM\_Config}+\frac{\sum_{i=1}^{w}\left(T_{i\_MAP}+T_{i\_REDUCE}\right)}{w}. \end{array}$$

From (12), it can be observed that *𝒯* (the computing time or makespan of BWASW-PMR) depends on the computation time of map and reduce worker nodes. The core alignment steps (based on the BWA-SW algorithm) are performed by the map worker nodes, that is, *𝒯*_REDUCE_ ≪ *𝒯*_MAP_. The time taken to initialize the map and reduce computing clusters based on VMs, that is, *𝒯*_VM_Config_, is dependent on the cloud platform considered for deployment and can be neglected. Therefore, it can be stated by the total makespan:

(13) $$\begin{array}{c} T\approx \frac{\sum_{i=1}^{w}\left(T_{i\_MAP}\right)}{w}. \end{array}$$

Optimizing the SW algorithm in BWA-SW is a possible solution to reduce the total makespan *𝒯*.

### 3.3. Smith-Waterman Algorithm Optimization

**Figure 4:**
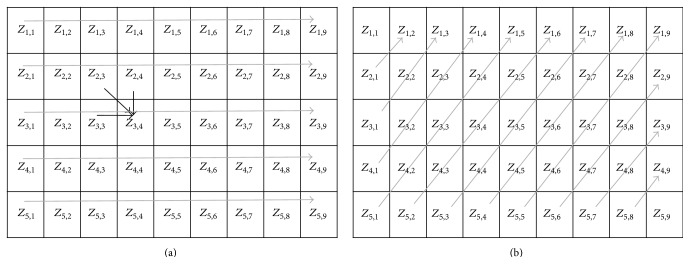
Smith-Waterman algorithm: (a) conventional computation technique and data dependencies and (b) wavefront based parallelized technique adopted in BWASW-PMR.

In the SW algorithm, computation of the similarity matrix score, that is, *Z*, requires the maximum time. Adopting a parallelization technique for computation of *Z* is considered in BWASW-PMR. Let us consider a query sequence *Q* and reference sequence *R*, whose sizes are 4 and 8, respectively. Therefore, the matrix *Z* to be computed is of size (5,9) and is shown in Figure 4(a). Data dependencies that exist in computing each element of *Z* make the parallelization technique difficult to implement. For computation of *Z*_3,4_ (shown as black lines in Figure 4(a)), it requires that the computation of *Z*_2,3_, *Z*_2,4_, and *Z*_3,3_ must be completed. Based on the sequences *Q* and *R*, and values of *Z*_2,3_, *Z*_2,4_, and *Z*_3,3_, the value of *Z*_3,4_ is obtained. The computation of *Z* is more often done serially as shown through the grey arrows in Figure 4(a). To parallelize the computation of *Z*, the adoption of CUDA/GPU based techniques was considered by researchers [33, 41]. However, the availability and cost of such computing platforms on public clouds are an issue. To maximize resource utilization available with the VM worker node at minimal costs in BWASW-PMR, wavefront based parallelization technique [42] is considered. The parallel computation technique adopted is shown by grey diagonal lines in Figure 4(b). Computation of all cells of *Z* that fall under the diagonal lines can be done in a parallelized fashion.

The parallelization technique adopted to compute *Z* in SW algorithm enables reducing the makespan of map worker nodes, that is, *𝒯*_MAP_.

## 4. Evaluation: Experiments

In this section, we study the performance of BWASW-PMR cloud platform. BWASW-PMR is developed using C++ and C#.Net and is deployed on the Azure cloud platform. BWASW-PMR adopts the BWA-SW algorithm with the optimization of SW algorithm. Experiments have been conducted to study the performance of the optimized SW algorithm. The performance of BWASW-PMR is compared with Bwasw-Cloud to perform long read alignments on the cloud platform. All genomic data considered for the experiments are obtained from NCBI database [3] that is publically available.

### 4.1. SW Optimization Analysis

Optimization of the SW algorithm in BWA-SW aligner for BWASW-PMR is a novel approach considering cloud deployment. To analyze performance of the optimized SW algorithm, comparison with the standard SW algorithm (hereafter referred to as the SW algorithm in this section) available within BWA-SW is considered. The optimization is achieved using a wavefront parallelization technique. The optimized version of SW is hereafter referred to as “SW-Optimized” in this section. Uniform SW parameter settings (i.e., gap penalty, matching, and mismatching score) are considered for analysis. For analysis, the sequences *R* and *Q* of equal lengths are considered. Equal length is considered to maximize the computations required for alignments. Section of* Homo sapiens* chromosome 15, GRCh38.p2 Primary Assembly (NC_000015.10), is considered as the reference *R*. Query sequences considered are obtained from the influenza virus database [43]. The query sequences considered are summarized in Table 1.

**Figure 5:**
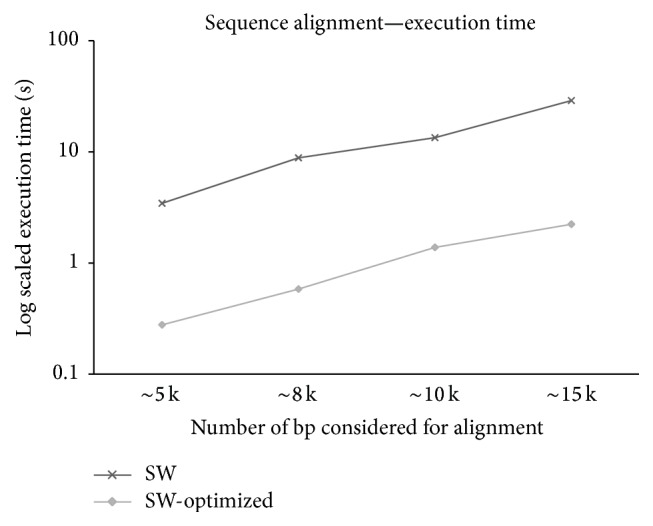
Execution time (in log scale—Base 10) of SW and SW-Optimized considering varied genomic sequence lengths.

Execution time of SW-Optimized and SW algorithm is noted for the four alignment pairs described. The results obtained are graphically shown in Figure 4. A logarithmic (Base 10) representation of the execution is considered in Figure 5. As sequence lengths for alignment are increased, the execution time of SW and SW-Optimized increases. The execution time of SW-Optimized is lower when compared to the considered serial SW algorithm. All experiments are executed on a Quad Core Intel i7 machine with 8 GB of RAM. An average reduction in the execution time of about 91.8% is observed. Results obtained prove SW-Optimized considered in BWASW-PMR outperforms the classical SW algorithm.

### 4.2. Experiments considering BWASW-PMR Cloud and Bwasw-Cloud Single Computing Node

**Figure 6:**
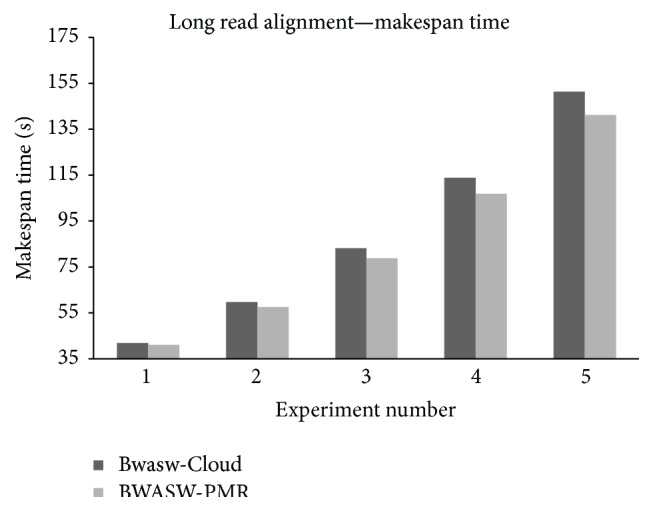
Makespan observations considering 5 long read sequence alignment experiments.

To evaluate the performance of BWASW-PMR, comparison with Bwasw-Cloud is considered. Deployments of Bwasw-Cloud and BWASW-PMR are considered with one computing node (i.e., one map worker and one reduce worker are considered). BWASW-PMR is deployed on the Azure cloud platform. Bwasw-Cloud is designed using the Hadoop framework. Apache Hadoop & YARN 2.4.0 version is used in the deployment of the Bwasw-Cloud. Uniform configurations of the computing nodes are considered in the deployments. Bakers yeast genomic database (i.e.,* Saccharomyces cerevisiae* S288c) is considered for evaluation [44]. Experiments using a reference genomic sequence and five query sequences of varied lengths are considered. The experiments conducted with the reference and query genomic sequences are summarized in Table 2. Makespan or total execution time is noted and the results obtained are shown in Figure 6. The results obtained prove that the proposed BWASW-PMR cloud aligner deployed on Azure outperforms Bwasw-Cloud deployed on Hadoop. In experiment 1, the speed-up achieved for BWASW-PMR is about 4.5. For longer sequence alignments, that is, in experiment 5, the speed-up was observed to be 7.5. As query length increases, the performance of BWASW-PMR improves. An average speed-up of 6.7 is achieved considering BWASW-PMR when compared to the Bwasw-Cloud.

### 4.3. Experiments considering BWASW-PMR Cloud and Bwasw-Cloud on Azure

**Figure 7:**
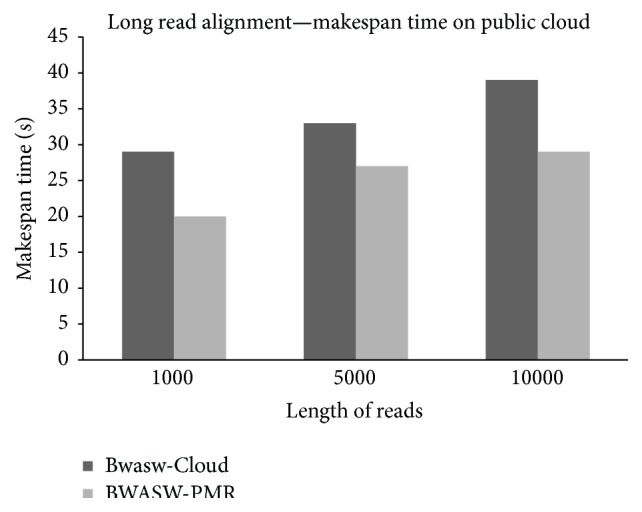
BWASW-PMR and Bwasw-Cloud makespan time comparisons for 150 reads of varied length considering the Azure public cloud deployments.

This section discusses public cloud deployments and performance analysis of BWASW-PMR against Bwasw-Cloud. Deployment of BWASW-PMR on the Amazon cloud and Azure cloud is possible. Here, deployment of BWASW-PMR on the Azure cloud is considered and presented. The deployed BWASW-PMR considers A3 VM instances. Each A3 VM instance consists of 4 computing cores, 7 GB of RAM, and 120 GB of local hard drive space. The deployment of Azure cloud consists of one master node and four worker nodes. HDInsight enables the deployment and provisioning of Apache Hadoop clusters on the Azure cloud platform [45]. Apache Hadoop & YARN version 2.6.0 is considered in the deployment of Bwasw-Cloud. Hadoop cluster of Bwasw-Cloud consists of 4 worker nodes of A3 VM instances and one master node.* Homo sapiens* chromosome 1 (NC_000001.11), consisting of approximately 250 million bp, is considered for evaluations. Overlapping of 10 k is considered. The query sequence segments are obtained from the* Homo sapiens* chromosome 1 segment reads. The length of the queries is varied (based on the read lengths) in terms of 1000 bp, 5000 bp, and 10000 bp. A total of 150 reads per length are considered. Generated log files obtained after the execution are studied to derive observations and results. The total makespan observed, that is, *𝒯*, is shown in Figure 7. The results prove that BWASW-PMR exhibits lower makespan time when compared to Bwasw-Cloud. As lengths of sequence reads increase, execution time increases due to the increase in alignment length sequences considered. Long sequence alignment considering BWASW-PMR gains a speed-up of 1.33 when compared to the Bwasw-Cloud aligner. The parallel executions of map and reduce phases along with SW optimization are the main contributing factors to the speed-up observed in this study.

**Figure 8:**
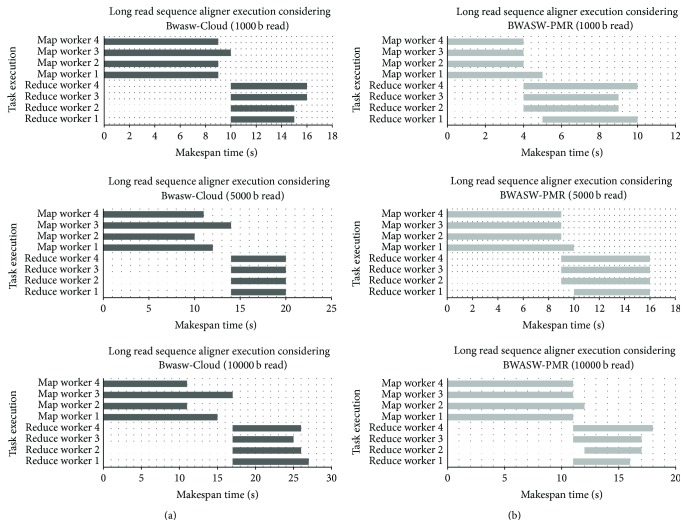
Long read sequence alignment, execution makespans of the map, and reduce worker nodes. (a) Bwasw-Cloud on Hadoop cluster of 4 nodes. (b) BWASW-PMR on Azure cluster of 4 nodes.

Detailed analysis of the experimental data reveals parallel execution of map function and SW optimization aid in reducing map makespans. The major alignment computations are carried out in the map phase, considering both BWASW-PMR and Bwasw-Cloud. An average reduction in the map phase makespan across all the experiments achieved by BWASW-PMR against Bwasw-Cloud is about 30%. The accumulation of the results, that is, aggregation of the alignment locations, is carried out in the reduce phase. No major computation is carried out in the reduce phase of BWASW-PMR and Bwasw-Cloud. The parallelization of the reduce function adopted in BWASW-PMR achieves an average reduction of 9.3% in *𝒯*_REDUCE_ when compared to Bwasw-Cloud. To prove efficiency in adopting a parallel map and reduce execution environment for the BWASW-PMR, makespans of the tasks executed at the map and reduce worker nodes are noted. The results obtained are shown in Figure 8. In Figure 8(a), the makespans of the worker nodes in Bwasw-Cloud deployed on the Hadoop cluster are shown. The makespans of the worker nodes in BWASW-PMR deployed on Azure are shown in Figure 8(b). From Figure 8, it is clear that the uniform tasks executed on BWASW-PMR exhibit lower makespans when compared to the execution on Bwasw-Cloud. In Figure 8(a), the reduce phase is initialized when all the map workers have completed their jobs. In BWASW-PMR, the reduce workers are running in parallel. The reduce phase is initiated when one or more of the map worker nodes have completed their jobs.

The SW optimization considered in BWASW-PMR reduces execution time of sequences to be aligned based on the SW algorithm. The experimental study and the results obtained prove that BWASW-PMR is capable of aligning long sequences using the cloud computing platform.

## 5. Comparison Notes with Related Work Counterparts

In this section, a comparison of BWASW-PMR with existing cloud aligners for long sequence reads is presented. Comparisons with Bwasw-Cloud [34], MapReduce-BLAST [33], CloudAligner [13], and the BWA-SW [10] aligners are considered. The BWA-SW sequence aligner [10] for long reads is memory hungry (high RAM requirements). Moreover, execution on the cloud platform is not considered. The CloudAligner [13] adopts the seed-and-extend algorithm for sequence alignment. The BWA-SW and BLAST also adopt similar approach. Though the seed-and-extend based aligners are fast, they suffer from low accuracy [10] and are more suitable for short read alignments. To achieve accurate alignment results considering long reads, the SW algorithm is adopted in the BWA-SW. MapReduce-BLAST [33] is a parallelized version of BLAST using MapReduce. Bwasw-Cloud bears the closest similarity to our work and is used for comparison in the experimental study presented here. Cloud-based aligners, that is, [13, 33, 34], consider the Hadoop framework for deployment. The drawbacks of the Hadoop framework have been discussed in the first section of the paper. To overcome these drawbacks, BWASW-PMR has been proposed in this paper. Comparison of BWASW-PMR with its related work counterparts is summarized in Table 3.

## 6. Conclusion and Future Work

Sequencing and analyzing of genomic data are important processes in bioinformatics. Rapid developments of the NGS technologies produce humongous amount of genomic data. For analysis of genomic data, alignment tools are used. The alignment tools are classified as short read type and long read type. The existing sequence aligners predominantly consider short read genomic sequences and lacking of support in cloud environment. These aligners exhibit deficiencies in the alignment of long sequence genomic data that are currently generated using NGS technologies. In NGS sequencers, users are required to wait for sufficient resources to become available in which the time needed to complete processing becomes unpredictable. More data is increasingly being generated which leads to serious issues in storing and processing. The existing long read aligners that adopt the cloud platform for computation suffer from drawbacks that are discussed in this paper. In this paper, we combined cloud infrastructure and MapReduce framework together as a solution to support long read sequence alignment. We proposed BWASW-PMR cloud platform to align long read sequences. The BWA-SW algorithm is adopted for long sequence alignment in BWASW-PMR cloud platform. Optimization of the SW algorithm and a Parallel MapReduce execution strategy are considered in BWASW-PMR. Parallel execution of the map and reduce functions is adopted to maximize resource utilization of the VM based cloud computing platform and reduce makespans. This paper highlights comparison of the proposed BWASW-PMR with the existing systems for long sequence alignments. The experiments presented have proven the efficiency of the optimized SW algorithm. Moreover, comparison with state-of-the-art Bwasw-Cloud for long sequence alignment is presented throughout the experimental study. The results obtained indicate significant improvement considering BWASW-PMR when compared to Bwasw-Cloud. Our proposed BWASW-PMR is of use to the genomic community to support the required computations for long sequence alignment efficiently. The parallel executions of map and reduce phases along with SW optimization are the main contributing factors to the speed-up observed in this study.

In the future, we propose to undertake optimization of the BWA-SW algorithm due to its memory hungry nature and also accelerating BWA-MEM algorithm of Burrows Wheeler aligner [12] on different platform.

## Figures and Tables

**Table 1:** Information of the genome sequences used as queries considering equal section lengths from the *Homo sapiens* chromosome 15 as reference.

Representation	Query genome definition (NCBI accession number )	Length
~5 k	Adelie penguin polyomavirus isolate AdPyV_Crozier_2012 (NC_026141.2)	4988 bp
~8 k	Rhesus monkey papillomavirus (NC_001678.1)	8028 bp
~10 k	*Xenopus laevis* endogenous retrovirus Xen1 (NC_010955.1)	10207 bp
~15 k	Japanese eel endothelial cells-infecting virus (NC_015123.1)	15131 bp

**Table 2:** Experiment information considered to compare the performance of BWASW-PMR with the Bwasw-Cloud.

Number	Reference genome (NCBI accession number)	Length	Query genome (NCBI accession number )	Length
1	TPA_inf: *Saccharomyces cerevisiae* S288c chromosome IV (BK006938.2)	1531933 bp	TPA_inf: *Saccharomyces cerevisiae* S288c chromosome V (BK006939.2)	576874 bp
2	TPA_inf: *Saccharomyces cerevisiae* S288c chromosome IV (BK006938.2)	1531933 bp	TPA_inf: *Saccharomyces cerevisiae* S288c chromosome XI (BK006944.2)	666816 bp
3	TPA_inf: *Saccharomyces cerevisiae* S288c chromosome IV (BK006938.2)	1531933 bp	TPA_inf: *Saccharomyces cerevisiae* S288c chromosome X (BK006943.2)	745751 bp
4	TPA_inf: *Saccharomyces cerevisiae* S288c chromosome IV (BK006938.2)	1531933 bp	TPA_inf: *Saccharomyces cerevisiae* S288c chromosome II (BK006936.2)	813184 bp
5	TPA_inf: *Saccharomyces cerevisiae* S288c chromosome IV (BK006938.2)	1531933 bp	TPA_inf: *Saccharomyces cerevisiae* S288c chromosome XVI (BK006949.2)	948066 bp

**Table 3:** Comparison of BWASW-PMR with its related work counterparts.

	BWASW-PMR	Bwasw-Cloud [34]	MapReduce-BLAST [33]	CloudAligner [13]	BWA-SW [10]
Long sequence alignment support	Yes	Yes	Yes	Yes	Yes
Alignment algorithm adopted	BWA-SW	BWA-SW	Blast	Seed-and-extend algorithm	BWA-SW
SW for accurate alignment	Yes	Yes	No	No	Yes
Cloud MapReduce platform execution support	Yes	Yes	Yes	Yes	No
MapReduce platform considered	Custom	Hadoop	Hadoop	Hadoop	No
SW optimization	Yes	No	No	No	Yes
Parallelization of MapReduce phases	Yes	No	No	No	No
Accuracy	Yes	Yes	No	No	Yes
